# Lignin-Based Admixtures: A Scientometric Analysis and Qualitative Discussion Applied to Cement-Based Composites

**DOI:** 10.3390/polym15051254

**Published:** 2023-03-01

**Authors:** Victor Rezende Carvalho, Laís Cristina Barbosa Costa, Bruno Eduardo Lobo Baeta, Ricardo André Fiorotti Peixoto

**Affiliations:** 1 Laboratory of Materials for Civil Construction, Federal University of Ouro Preto, Ouro Preto 35400-000, Brazil; 2 Laboratory of Technological and Environmental Chemistry, Federal University of Ouro Preto, Ouro Preto 35400-000, Brazil

**Keywords:** lignin-based admixtures, lignin valorization, cement-based composites, sustainable development, scientometric analysis

## Abstract

The development of lignin-based admixtures (LBAs) for cement-based composites is an alternative to valorizing residual lignins generated in biorefineries and pulp and paper mills. Consequently, LBAs have become an emerging research domain in the past decade. This study examined the bibliographic data on LBAs through a scientometric analysis and in-depth qualitative discussion. For this purpose, 161 articles were selected for the scientometric approach. After analyzing the articles’ abstracts, 37 papers on developing new LBAs were selected and critically reviewed. Significant publication sources, frequent keywords, influential scholars, and contributing countries in LBAs research were identified during the science mapping. The LBAs developed so far were classified as plasticizers, superplasticizers, set retarders, grinding aids, and air-entraining admixtures. The qualitative discussion revealed that most studies have focused on developing LBAs using Kraft lignins from pulp and paper mills. Thus, residual lignins from biorefineries need more attention since their valorization is a relevant strategy for emerging economies with high biomass availability. Most studies focused on production processes, chemical characterizations, and primary fresh-state analyses of LBA-containing cement-based composites. However, to better assess the feasibility of using different LBAs and encompass the multidisciplinarity of this subject, it is mandatory that future studies also evaluate hardened-sate properties. This holistic review offers a helpful reference point to early-stage researchers, industry professionals, and funding authorities on the research progress in LBAs. It also contributes to understanding the role of lignin in sustainable construction.

## 1. Introduction

Lignin is the second most abundant polymer in nature and can be isolated from wood, annual plants, or agricultural residues by different processes [1]. The global generation of lignin is estimated at 100 million tons/year, attributed to biorefineries and pulp and paper mills [2,3]. Due to their variety of functional groups (such as hydroxyl, carboxyl, and methoxyl), lignin can be applied as raw material in resins, fibers, fillers, adhesives, and dispersing agents, offering different possibilities for the industry [4]. However, despite their high availability and potential for application, lignins obtained from industrial processes, known as technical lignins, are usually treated as by-products and underutilized [5,6].

The main delignification methods are the Sulfite, Kraft, Soda, and Organosolv processes [7,8]. These processes generate a waste by-product called black liquor, from which technical lignins are isolated. Lignosulfonates represent the technical lignins obtained from the sulfite process and are widely commercialized as low-cost admixtures for cement-based composites [3]. The dispersing properties of lignosulfonate favor the water consumption reduction in cement mixtures, which results in a more densified and homogeneous matrix with higher mechanical strength and durability [9,10]. Despite the mentioned properties, the limited worldwide production and non-standardization of performance and composition restrict the use of commercial lignosulfonate-based admixtures, which motivates the research on new lignin-based admixtures (LBAs) [11,12]. The production of LBAs from residual lignins contributes to waste management in biorefineries and pulp and paper mills, in addition to being an alternative to reduce the commercialization of petroleum-based admixtures [13,14]. The construction sector is responsible for pollutant emissions and high consumption of natural resources [15,16]. In this sense, it is mandatory to develop studies seeking to reduce these environmental impacts, such as LBAs incorporation on cement-based composites.

The LBAs developed so far were classified as plasticizers [17], superplasticizers [18], set retarders [19], and air-entraining admixtures [20]. These new LBAs can be produced by modifying commercial lignosulfonate-based admixtures [21,22] or using other technical lignins [23,24]. Different modification methods can be applied to lignins to improve the efficiency and mechanisms of action of LBAs, which include copolymerization with other compounds and an increase in sulfonic groups, molecular weight, and polarity [25]. The structure and composition of lignins vary according to the botanical species and the delignification process employed [3,26], which also influences the characteristics and application possibilities of LBAs. In addition, LBAs can be produced from unmodified technical lignins and black liquors [27,28], avoiding further modification strategies.

### 1.1. Characteristics of Technical Lignins

Technical lignins are those produced in industrial delignification processes. They are mainly generated in pulp and paper mills, although the lignin generation during biomass pretreatment in lignocellulosic biorefineries has been increasing in recent years [29,30]. Despite their high availability and applicability in industry, lignins from pulp and paper mills have been used on a large scale as fuel for heat and electricity, with less than 2% being used in the production of specialty chemicals [2,3,6]. Regarding residual lignins from biorefineries, it is still necessary to create better strategies for their commercialization [31]. It is worth mentioning that the inappropriate disposal of residual black liquors can cause serious environmental problems [32].

In the literature, lignins are divided into sulfur-containing lignins and sulfur-free lignins. The Soda and Organosolv processes generate sulfur-free lignins, while the Sulfite and Kraft processes generate sulfur-containing lignins [8,9]. Lignosulfonates are generated in the sulfite process and have a high molecular weight (1000–120,000 g/mol) and water solubility, which contributes to their commercial application [33]. In general, the sulfur content contributes to the higher solubility and anionic charge density of sulfonated lignins, as the molecular weight is directly related to the adsorption characteristics of these compounds [34,35].

Kraft lignins have lower sulfur content and lower molecular weight (1500–25,000 g/mol) compared to lignosulfonates [33]. The Kraft process is widely used in pulp and paper mills. The high availability and underutilization of residual Kraft lignins are some of the reasons that justify the interest of researchers in their valorization [5,34]. The Soda process is typically applied in the delignification of agricultural crops [2]. Soda lignins have higher purity and lower molecular weight than lignosulfonates (1000–15,000 g/mol) [8,33]. Finally, Organosolv lignins are those obtained from the homonymous delignification process, which involves the use of organic solvents at high temperatures. These lignins have high purity, low molecular weight (500–5000 g/mol), and low water solubility, although their characteristics depend on the solvents used [2,33]. It stands out that procedures that apply a green chemistry approach using eco-friendly solvents are currently preferable [36]. More in-depth information about production, characteristics, and applications of technical lignins can be found in existing works [3,5,26].

### 1.2. Lignosulfonate-Based Admixtures

The worldwide production of lignosulfonates is about 1 million tons/year [37]. Lignosulfonate is obtained from the black liquor generated during the paper manufacturing process. It is composed of 20–30% lignin and contains free sulfurous acid and sulfates, decomposition products of cellulose and lignin, different carbohydrates, and a complex mixture of sulfonation products of lignin [38]. In the construction industry, lignosulfonates are widely commercialized as 1st generation admixtures. Among the different types of lignosulfonate-based admixtures available are those composed of sodium, potassium, calcium, and magnesium salts [39]. Depending on the associated cation (Ca^+2^, Na^+2^, Mg^+2^, etc.), pH values may vary among these admixtures (pH between 4.0 and 9.0), although their performance is not affected [40].

**Figure 1:**
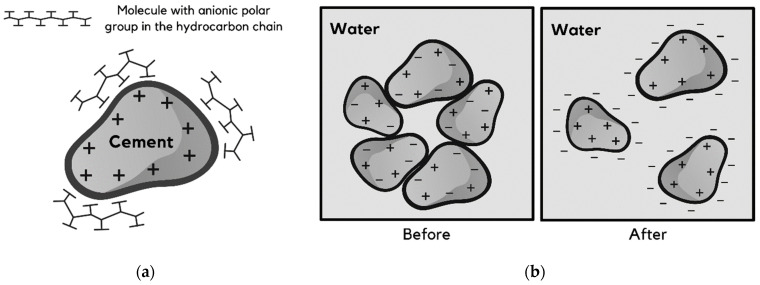
(**a**) Adsorption of the polar chains of a surfactant admixture on the surface of the cement particle; (**b**) Floc formation by cement particles before the incorporation of a surfactant admixture, and dispersion of flocs after the incorporation (adapted from Mehta and Monteiro [41]).

The molecular structure of lignosulfonates has a significant influence on their plasticizing properties. When incorporated into cement-based composites, their polar chains with anionic functional groups, such as sulfonic acids (SO_3_H), are adsorbed onto the cement particles. As a result, the polar end of the lignosulfonate is directed towards the water, which reduces its surface tension and makes the cement particle hydrophilic [41]. Although electrostatic repulsive force is the primary mechanism of lignosulfonate-based admixtures, their performance may also be associated with steric hindrance in some cases [35]. On the other hand, 3rd generation chemical admixtures, such as polycarboxylate-based superplasticizers, have a high steric repulsion effect that enhances their plasticizing abilities [42]. Figure 1 illustrates the mechanism of action of lignosulfonate-based and other surfactant admixtures.

Besides being classified as plasticizers or water reducers, lignosulfonate-based admixtures can act as air-entraining and set-retarding agents in cement-based composites [39]. In most cases, their influence on different properties of cement matrices can be seen as a negative point for their application [11,39,40]. The retarding effect of lignosulfonates is related to the presence of sugars (such as glucose and sucrose) in their chemical composition, which may interfere with cement hydration [43]. Other disadvantages of lignosulfonate-based admixtures are related to commercial aspects: (1) the Kraft pulping process is more applied than the Sulfite process, limiting the global production of lignosulfonate [44]; (2) Lignosulfonates have high variability in composition and purity [11,33]; (3) Lignosulfonates have a limited amount of sulfonate groups that interfere with the anionic charge density of their polymer chains [12].

### 1.3. Research Aim

The development of new LBAs contributes to the sustainable management of natural resources and to the improvement of the properties of cement composites. Therefore, the importance of a study that gathers existing data in the literature and manages to demonstrate the importance of LBA for chemical engineers, materials engineers, agronomists, civil engineers, and other professionals, is remarkable. Through a scientometric analysis and a qualitative discussion, this study aims to highlight the current state of LBAs research applied to cement-based composites and critically review the existing works. The proposed review strategy allowed the authors (1) to identify publication trends, most frequently studied terms and keywords, most relevant publication sources, and most influential and collaborative scholars and countries in LBAs research; (2) to analyze the studies found in the literature and discuss the main characteristics of new LBAs incorporated in cement mixtures; (3) to identify current knowledge gaps and propose new possibilities for near-future research on LBAs applied to cement-based composites.

As far as we know, no work exists in the literature that presents a scientometric approach and a qualitative discussion on the subject that covers so many articles, proving the novelty of the present work. Previous studies mentioned the application of LBAs in cement-based composites [44,45]. However, the reviews published so far focused on manufacturing routes and chemical characteristics of lignin-based materials, not emphasizing the applicability of LBAs in cement mixtures. In this sense, the proposed scientometric review quantitatively addresses the main parameters of LBAs research, while the qualitative discussion presents the current research scenario as a reference point to early-stage scholars. This holistic review will help the specialized scientific community to better understand the evolution of the theme over time, the existing weaknesses so far, future expectations, and existing bottlenecks for the application of LBAs on a larger scale. In addition, this study highlights LBAs as an alternative to producing more efficient and sustainable cement composites.

## 2. Methodology

This study has two stages: science mapping and qualitative discussion. The science mapping studies employ three techniques: bibliometric analysis, scientometric analysis, and informatics. The bibliometric analysis seeks to map the literature per se, while scientometrics extends the investigation to literary production, establishing connections between researchers, affiliations, and countries [46,47]. In this research, the authors applied a scientometric analysis for science mapping.

The second stage refers to the qualitative discussion of the data based on the analysis of the documents found in the first stage. The authors expect that science mapping helps to construct a consistent literature review that facilitates the decision-making for future researchers on the subject. In addition, previous studies developed a science mapping analysis followed by a more in-depth discussion of the literature about other themes [48,49,50].

**Figure 2:**
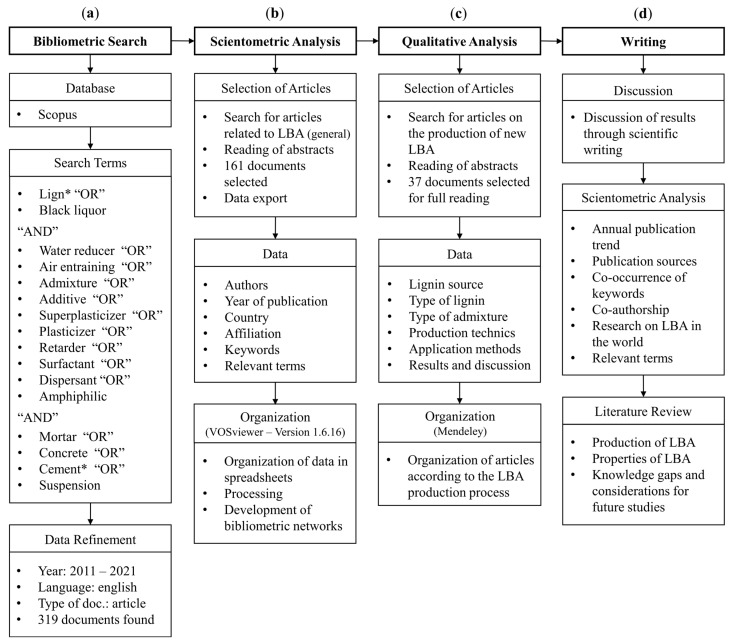
Stages of science mapping and literature review: (**a**) Bibliometric Search; (**b**) Scientometric Analysis; (**c**) Qualitative Analysis; (**d**) Writing. The asterisk symbol and the commands “AND” and “OR” were used in Scopus to expand and organize the bibliometric search, respectively.

Figure 2 presents in detail the stages of the review strategy. This scheme summarizes the search parameters and research tools adopted, as well as the aims of this study.

### 2.1. Search Tools

The bibliometric search was performed in Scopus because of its wide range of journals and up-to-date articles compared to other databases [51]. The Scopus database allows one to export files to Mendeley, a free reference management software. It is important to point out that Scopus is compatible with bibliometric analysis software. The documents found were analyzed in VOSviewer (version 1.6.16), an open-access software developed exclusively bibliometric networks analyses [52]. This text-mining tool enables the creation, visualization, and exploration of maps based on network data, such as journals, scholars, affiliations, countries, keywords, and terms [53]. These data can be organized by co-authorship, co-occurrence, citation, bibliographic coupling, and co-citation networks [53].

Recent studies focused on the civil construction sector developed a scientometric analysis of documents filtered in Scopus using VOSviewer [54,55,56]. Thus, the tools adopted were consistent with the proposals of this study.

### 2.2. Bibliometric Search

Figure 2a shows the advanced search parameters adopted in Scopus searched within titles, abstracts, and keywords. The authors decided to include the term “black liquor”, which refers to the by-product of delignification processes. In addition, the term “suspension” was adopted to expand the search for lignin-based admixtures (LBAs). A total of 958 documents met the initial restrictions. After the data refinement, 319 articles were selected. A further filtering step was carried out by reading the abstracts of the 319 selected articles. The authors considered the following topics: (1) evaluation of commercial lignosulfonates properties; (2) production of new LBAs for different suspensions and composites; and (3) production of new LBAs for cement-based composites. Finally, 161 articles met all the restrictions. It is recommended to generate maps with at least 100 items to ensure the usefulness of VOSviewer resources [57]. Therefore, the number of documents found was appropriate for the proposed scientometric analysis.

### 2.3. Scientometric Analysis

Figure 2b indicates the criteria adopted for the scientometric analysis. Initially, the results provided by the Scopus analyser were used to describe the annual publication trend. Subsequently, the bibliometric data of the 161 articles were downloaded and imported into the VOSviewer. Therefore, the data collection and map generation were performed using the software.

Initially, the VOSviewer functionality “Create a map based on bibliographic data” was selected. The scientific mapping of most relevant journals for the topic was carried out by setting the “Type of analysis” and the “Unit of analysis” to “Citation” and “Sources”, respectively. Mapping these journals allows the analysis of existing research trends and presents alternatives for researchers. The requirement of a minimum number of publications per journal was 3. As a result, 13 out of 73 journals met the threshold.

The analysis of the co-occurrence network of keywords was performed by setting the “Type of Analysis” to “Co-occurrence”, the “Unit of Analysis” to “Author Keywords”, and the “Counting Method” to “Fractional Counting”. Keywords reflect the theme of research publications and are necessary for indexing articles in databases [54]. Accordingly, 39 out of 419 keywords were selected, considering terms with at least 3 occurrences.

To identify most relevant scholars, the “Type of Analysis” and “Unit of Analysis” were set to “Co-authorship” and “Authors”, respectively. The scientific collaboration networks analysis facilitates access to funds, specialties, and expertise. In addition, it contributes to productivity and prevents investigators from isolation [47]. A search for authors with equivalent names was previously carried out to avoid misinterpretation of data. The minimum number of documents per author was set to 3. Consequently, 28 out of 433 names were identified and 24 authors were selected by analyzing name overlaps.

The “Unit of Analysis” was set to “Countries” to identify the most collaborative countries in LBAs research. Determining the most collaborative countries on a research theme facilitates the establishment of new partnerships and joint research funding programs. In addition, it aids in promoting the exchange of technologies and innovation [54]. Publications from 40 countries were identified, mainly from Europe, Asia, and North America. The minimum number of documents and citations per country was set to 3 and 10, respectively. Therefrom, 15 countries met the thresholds.

Finally, the VOSviewer functionalities “Create a map based on text data” and “Read data from bibliographic database files” were selected. The terms were extracted from “Titles and Abstract Fields” to identify most relevant terms in LBAs research. Subsequently, the “Counting Method” was set to “Binary Counting” and the minimum number of occurrences of each term was set to 10. The analysis of most relevant terms provides a more comprehensive approach to scientific publications and complements the evaluation of the co-occurrence network of keywords. As a result, 56 out of 3509 terms met all the restrictions.

### 2.4. Qualitative Analysis

The second stage of data analysis was performed as indicated in Figure 2c. The article selection process considered the production and characterization of new LBAs for cement-based composites. As a result, 37 articles met the threshold and were read for a further in-depth discussion. The discussion divided the existing research into categories, based on the characteristics of the LBA. After that, the authors describe the existed findings and knowledge gaps, leading to new research topics and directing future work.

## 3. Results

### 3.1. Annual Publication Trend

**Figure 3:**
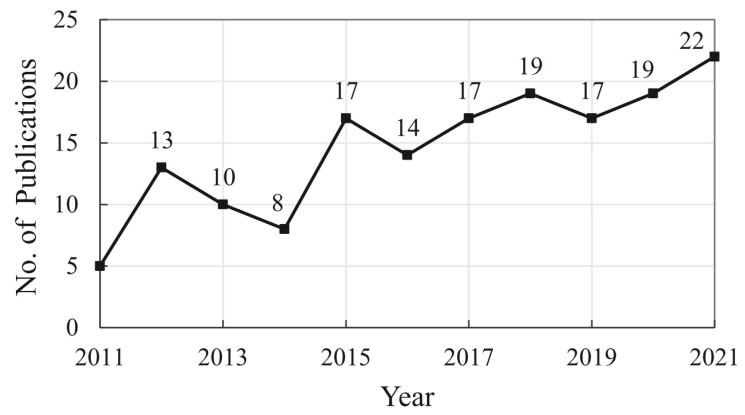
Annual publication trend in LBAs research from 2011 to 2021.

Figure 3 presents the annual publication trend in the LBAs research between 2011 and 2021. The number of publications considered the literature published up to January 2022. Therefore, publications in 2021 are fully counted.

Although there is some fluctuation in data, the LBAs research has witnessed growth over the 11 years. The increase between 2011 and 2018 is related to the higher participation of Chinese and North American scholars in LBAs research. China, Canada, and the United States were responsible for about 47% of the selected publications between 2011 and 2018. This trend has strengthened over the years, and the collaboration between European, Asian, and North American countries is mainly responsible for the high number of publications between 2019 and 2021.

The interest of scholars in LBAs research is a consequence of the current economic scene. With the enhancement of the bioeconomy, the global lignin market has been expanding [37]. North American and European companies have been increasing their investments to transform lignin into a more attractive raw material for the market through new lignin-based products [13]. In addition, there is a growing demand for the lignin market in emerging Asian economies such as China, Japan, India, and Indonesia [3].

### 3.2. Analysis of Publication Sources

**Figure 4:**
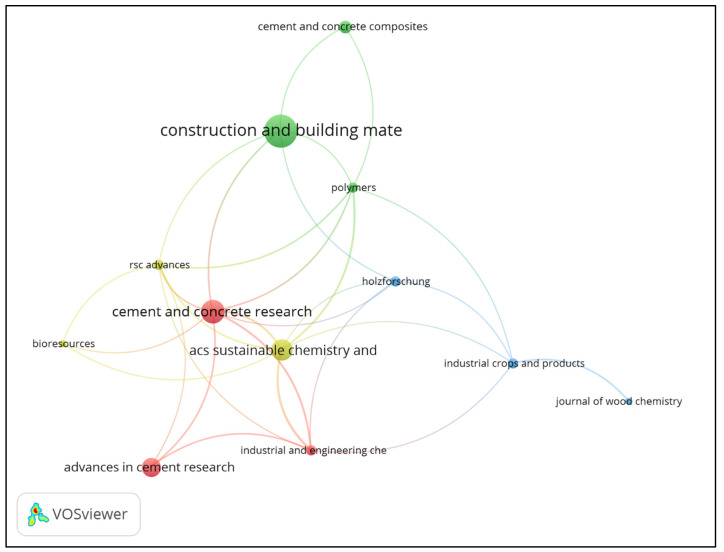
Science mapping of publication sources.

Figure 4 presents the network of publication sources in the LBAs research. The size of each node is related to the number of publications in the journals. In addition, the journals are grouped into 4 clusters (separated by color). The clusters suggest that those journals have a higher degree of interconnection (in terms of citation). Thus, articles published in journals from the same cluster tend to be mutually cited. Therefore, the citations from *Polymers* are more correlated with *Construction and Building Materials* and *Cement and concrete composites*.

Table 1 shows the most relevant journals in LBAs research, organized according to the number of published articles. *Construction and Building Materials* is the scientific journal with the highest number of publications in the LBAs research, which justifies the size of the node shown in Figure 4. *Construction and Building Materials* also presents a significant number of citations and average normalized citations. The normalization parameter corrects the fact that older articles have had more time to be cited and, therefore, may have a higher number of citations when compared to more recent articles [53].

*Cement and Concrete Composites* presents a relatively low number of publications but expressive average normalized citations. Thus, the high number of annual citations from *Cement and Concrete Composites* indicates its contribution to LBAs research. In addition, Table 1 shows that *Cement and Concrete Research* has the highest total link strength. The total link strength is an indicator of the correlation network of journals. *ACI Materials Journal* has a total link strength of 0, so it is not presented in the science mapping of publication sources (Figure 4).

Scientific journals with different categories publish about LBAs. As a result, scientific journals on engineering, environmental engineering, agricultural and biological sciences, materials engineering, chemical engineering, chemistry, and materials science were identified. The multidisciplinarity of LBAs research has a notable impact on the studies found in the literature and will be discussed later.

### 3.3. Co-Occurrence Network of Keywords Analysis

**Figure 5:**
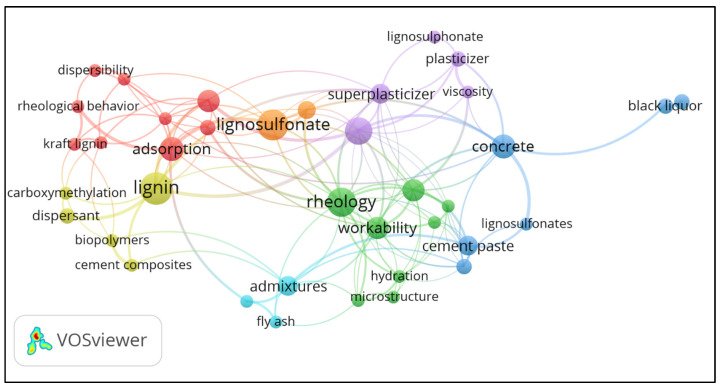
Co-occurrence network of keywords.

Figure 5 presents the most relevant keywords studied in LBAs research and their inter-relatedness. The size of each node is related to the number of occurrences of the keywords.

The keywords “rheology”, “adsorption”, “concrete”, “lignin”, and “lignosulfonate” stood out. These keywords are located in different clusters, so they co-occur more often with other keywords. In all, the keywords were grouped into 7 clusters. The keyword adsorption co-occurred most frequently with “charge density”, “dispersibility”, “dispersion”, “Kraft lignin”, “molecular weight”, “rheological behavior”, “sulfomethylation”, and “zeta potential”. On the other hand, the keyword lignin co-occurred more frequently with “dispersant”, “cement composites”, “carboxymethylation”, and “biopolymers”.

Table 2 complements Figure 5 and shows the most relevant keywords in LBAs research, ordered according to their total link strength.

The keywords “cement” together to “concrete” and “cement paste” refer to cementitious materials. It is relevant to point out the presence of the keywords “workability”, “adsorption”, and “dispersion”, which are phenomena observed in cement-based composites and different suspensions in the presence of chemical admixtures. In addition, 10 of the 15 most relevant keywords studied in LBAs research were adopted in the bibliometric search, indicating a good choice of search terms. There is some correlation between the number of occurrences and the total link strength, suggesting that a keyword with a high number of citations is more likely to co-occur with other frequently cited keywords.

### 3.4. Co-Authorship Network Analysis

**Figure 6:**
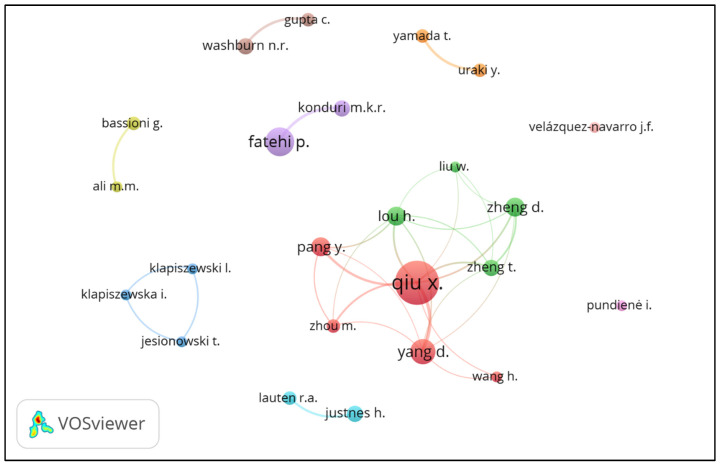
Co-authorship networks of collaborative scholars.

Figure 6 shows the co-authorship networks in the LBAs research. The size of each node is related to the total link strength of the authors.

There is a more comprehensive cluster of collaborative researchers formed by: Qiu X., Pang Y., Yang D., Zheng T., Zheng D., Zhou M, and Lou H. Their collaboration is regional, within the territory of Guangzhou (China), between the Guangdong University of Technology and the South China University of Technology. Again, Klapiszewski L., Klapiszewska I., and Jesionowski T. constitute one cluster of collaborative researchers. There are five other clusters composed by only two authors: Fatehi P. and Konduri M.K.R.; Lauten R.A. and Justnes H.; Ali M.M. and Bassioni G.; Washburn N.R. and Gupta C.; and Yamada T. and Uraki Y.

Table 3 shows the numerical indices of the co-authorship network. The researchers are ordered according to the number of documents published. The average of normalized citations represents the total number of citations by each author in a given average year.

The displayed data confirm the relevance of Qiu X. in the LBAs research. The set of 20 documents attributed to Qiu X. includes studies on the modification and use of lignin as an admixture for cement-based composites [18,20,58,59] and as a dispersing agent for graphite [60] and carbendazim [61] suspensions. Another relevant contributor to LBAs research is Fatehi P., with 11 publications. The collaboration between Fatehi P. and Konduri M.K.R. should also be noted. Fatehi P. and Konduri M.K.R. develop research at the Lakehead University (Thunder Bay, ON, Canada) and have published studies on the production of admixtures for cement-based composites [62], dispersants for coal [63], clay [23], and kaolin [64] suspensions. The partnership between Washburn N.R. and Gupta C. is also relevant in the LBAs research. Washburn N.R. and Gupta C. develop research at the Carnegie Mellon University (Pittsburgh, PA, USA) and have published studies on the performance of LBAs in unmodified cementitious suspensions [65], and in cementitious suspensions with kaolin clay and zeolite [66] and simulated with magnesium oxide [67]. Figure 6 also shows another 6 unconnected co-authorship networks.

Figure 6 and Table 3 show that co-authorship networks were restricted by setting the number of documents per author to 3. Although Fatehi P. has 11 published articles, the total link strength attributed to him is limited to the works in partnership with Konduri M.K.R. (5 co-authorships). In the same way, Pundienè I. and Velázquez-Navarro J.F. have a total link strength equal to 0, even though they have met the restrictions of at least 3 published documents. These unconnected authors occur because none of their collaborators complied with the restrictions adopted, which justifies their isolated names in Figure 6.

Depending on the measure of productivity, the ranking of the researchers differs significantly. The most cited authors in the LBAs research include Qiu X., Fatehi P., Yang D., Konduri M.K.R., and Pang Y. Considering the criteria of average normalized citations, the most influential authors include Konduri M.K.R., Velázquez-Navarro J.F., Fatehi P., Wang H., and Yang D. The relevant authors in LBAs research are mostly chemists, chemical engineers, and materials engineers.

### 3.5. Active Countries in the LBA Research

**Figure 7:**
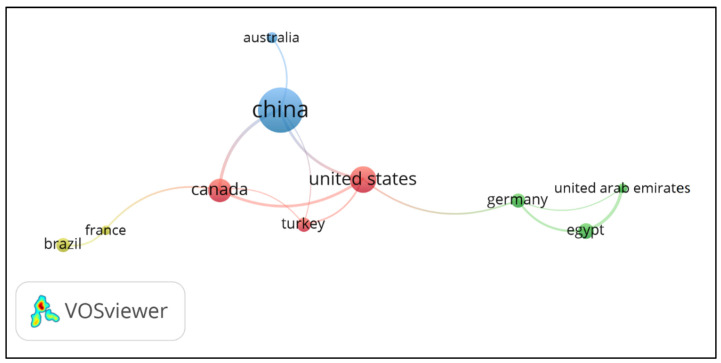
Most contributing countries in the LBAs research.

Figure 7 presents the most relevant and collaborative countries in the LBAs research. The size of the nodes represents the relevance of the countries to the subject. The network is grouped into 4 main clusters. The clusters are not necessarily related to the geographic proximity of the countries.

Table 4 organizes the most relevant countries numerically and complements the information presented in Figure 7. Japan, Lithuania, Norway, Spain, and India do not appear on the network map. These countries had a total link strength equal to 0, which means that their collaborating countries did not comply with the restrictions adopted.

China stands out in terms of the number of publications and citations. China’s interest in LBAs research is a consequence of its large production of residual lignin due to the paper industries [58]. The total emission of black liquor in China is approximately 3.2 million tons per year, representing about 16% by mass of all wastewater from the industrial sector [20]. In addition to pulping and paper mills residues, Chinese researchers are also studying lignins generated in biorefineries to develop new admixtures [31]. Along with China, Japan and India are the main agents in the manufacture of lignin-based products in Asia [3], which justifies the interest of Japanese and Indian researchers in LBAs.

Based on the number of articles published, the most relevant countries in the LBAs research include China, USA, Canada, and Spain. Considering the criteria of average normalized citations, the major influential countries include Australia, Spain, France, and Canada. The relevance of the USA and Canada in the LBAs research may be related to the growing interest of North American companies in lignin-based products. The same happens with European countries, such as Spain, Norway, Turkey, and Germany. Brazil appears among the most collaborative countries in the LBAs research as the only representative of South America. The studies carried out by Brazilian researchers are strongly related to agro-industrial residues and have become popular in recent years. Although studies from the five continents were identified, African countries participated less in LBAs research. Only 4 out of the 40 identified countries are from Africa, with Egypt accounting for 70% of the total publications and 67% of the total citations.

### 3.6. Relevant Terms in the LBAs Research

Table 5 shows the top 20 most relevant terms in the LBAs research, according to their relevance score. The relevance score is calculated to exclude general terms [53].

The most frequent terms were “cement” and “lignin”. The most relevant term was “FTIR”, an acronym for Fourier-transform Infrared Spectroscopy. FTIR is a characterization technique widely used in studies on lignin-based products that provide important information on the structure of lignins, and other organic compounds. The presence of “Kraft lignin” among the most relevant terms is probably due to the higher number of studies on this technical lignin. It is also important to note the term “molecular weight”, which is a relevant parameter in the characterization of sulfonated lignins and will be analyzed in the qualitative discussion. The “zeta potential” is a parameter used to assess the stability of suspensions and is related to the repulsion or attraction of particles. Finally, the terms “workability”, “rheology”, and “yield stress” refer to the fresh-state behavior of pastes, mortars and concrete under different strains, and stresses.

In general, all terms found were consistent with the research subject. It stands out that 6 of the 20 most relevant terms were originally used in the advanced search in the Scopus database. In addition, the analysis of the most relevant terms brought important characterization parameters to the discussion on LBAs, which did not occur in the evaluation of most studied keywords.

## 4. Qualitative Discussion

Table 6 summarizes the information from the studies discussed on this topic. The authors used “not available” in the table to denote information not supplied by the reference paper.

LBAs are developed from different botanical species and production methods, which could include lignin modification processes. Table 6 also shows that LBAs can be incorporated into cement-based composites with mineral admixtures and their dosage ranges can vary significantly depending on the product’s characteristics. Finally, most LBAs identified in the literature are classified as plasticizers or water reducers. However, some LBAs were assorted as hybrid admixtures, air-entraining agents, grinding aids, and set retarders.

### 4.1. Development of New LBAs and Their Influence on Workability

The development of new LBAs from unmodified technical lignins and black liquors can reduce production costs, enabling large-scale commercialization. El-Mekkawi et al. [27] evaluated the black liquor obtained from the Soda pulping of rice straw in a pulp and paper mill. The replacement of 15% of water volume by black liquor in concrete with a water/cement ratio of 0.4 contributed to an increase of 105 mm in the Slump Test. In another study, Akond and Lynan [82] evaluated a lignin extracted from sugarcane bagasse by deep eutectic solvents (a mixture of formic acid and choline chloride was used in the study). The authors observed that a lignin incorporation of 0.3 wt.% contributed to an increase of approximately 50% in the mini-slump spread of a cement paste with a water/cement ratio of 0.47.

Studies conducted by Klapiszewski and collaborators also demonstrated that unmodified lignins could replace commercial lignosulfonate to develop hybrid (organic–inorganic) admixtures for cement-based composites [84,85,86,87]. In addition, these authors observed that a 1.0 wt.% incorporation of hybrid admixture with an alumina/lignin ratio of 0.2 increased the spread of the cement paste by 21% [85]. Using the same dosage for the hybrid admixture but with a silica/lignin ratio of 0.2, the spread of the cement paste enhanced by 7% [86]. In both studies, the pastes containing only lignin showed even better workability.

In addition to applying unmodified technical lignins and black liquors, different lignin modification processes for LBAs production have been reported in the literature. As synthesized by Alwadani and Fatehi [88], hydrophilic functional groups can be introduced into the hydrophobic tail of lignin to form: (1) anionic polymers, by introducing carboxylates, sulfates, and sulfonates [19,58]; (2) cationic polymers, by reacting with ammonium salts [20]; and (3) non-ionic polymers, by reacting with alcohols (polyoxyethylenated chains) [74].

Sulfonation and sulfomethylation processes were studied by different researchers [31,58,62,68,69]. These processes usually involve the addition of sodium sulfite and formaldehyde [89], which are responsible for introducing sulfonate and methyl groups into the lignin structure, respectively [62]. The plasticizing ability of sulfonated and sulfomethylated lignins is attributed to their molecular weight and anionic charge density [34]. The introduction of sulfonic groups increases these parameters (molecular weight and anionic charge density) and improves the adsorption capacity of the modified lignins in the cement particles [31]. Thus, the Zeta potential of the system enhances, indicating a higher electrostatic repulsion between the cement particles [24].

Complementary processes of oxidation by peracetic acid [22], hydrogen peroxide [14], and nitric acid [12] to increase the efficiency of sulfomethylation have been reported in the literature. In summary, oxidation processes enhance the reactivity of lignins by favoring the introduction of more sulfonic groups into their polymeric structures during sulfomethylation [34]. He and Fatehi [12] evaluated the performance of a softwood kraft lignin modified by oxidation and sulfomethylation in pastes with a water/cement ratio of 0.35. The incorporation of 0.4 wt.% of oxidized and sulfomethylated lignin increased the spread of the cement paste by 167% compared to the unmodified Kraft lignin and 40% compared to a commercial sodium lignosulfonate [12]. In another study, Yu et al. [22] evaluated the performance of a commercial sodium lignosulfonate modified by oxidation and sulfomethylation in pastes with a water/cement ratio of 0.4. As a result, adding 0.3 wt.% of the oxidized and sulfomethylated lignosulfonate, the spread of the cement paste increased by 15% compared to the paste containing the unmodified lignosulfonate [22]. In both studies, the molecular weight and the degree of sulfonation of the lignins enhanced due to the modification processes adopted.

Oxidation processes without sulfomethylation were also adopted for the development of new LBAs [70,71]. Kalliola et al. [70] evaluated the performance of a wheat straw Soda lignin modified by alkali-oxidation when added to mortar and concrete mixtures. As a result, the incorporation of 0.4 wt.% of the oxidized lignin resulted in a higher concrete slump and mortar spread when compared to a commercial lignosulfonate. Furthermore, the oxidized lignin performed similarly in the slump test to a commercial polycarboxylate-based superplasticizer evaluated at 0.2 wt.% content. The alkali oxidation was also responsible for an increase in the molecular weight of the lignin. Despite the relevance of molecular weight to evaluating sulfonated and oxidized lignins, this parameter can vary considerably concerning the type of lignin and the modification method adopted (see Table 6). Moreover, high molecular weight and sulfur content can prejudice the dispersion properties of LBAs [34].

In addition to sulfonation and oxidation processes, different polymerization methods have been reported for new LBAs production. In general, polymerization methods allow the development of more technological LBAs, with mechanisms of action similar to those of polycarboxylate-based superplasticizers. These admixtures consist of copolymers composed of methacrylic acid and poly(ethylene glycol) methacrylate [73]. The plasticizing ability of polycarboxylate-based admixtures results from the mechanisms of: (1) electrostatic repulsion, due to the adsorption of anionic groups (e.g., carboxylic groups) from the main chain on the cement particles; (2) stabilization of the system by steric hindrance, promoted by ether groups present in neutral side chains mostly composed by poly(ethylene oxides) [42,90].

The research group formed by Gupta, Washburn, and collaborators developed sustainable superplasticizers by grafting hydrophilic polyacrylamide onto Kraft lignins by controlled radical polymerization [65,66,67]. Gupta et al. [65] evaluated the performance of polyacrylamide-modified lignin in pastes with a water/cement ratio of 0.42. At dosages of up to 0.15 wt.%, the modified lignin showed better efficiency in the Slump test than a commercial polycarboxylate-based superplasticizer. These authors identified that the charges present in the lignin core and the side chains of the grafted polymer promoted steric and electrostatic interactions between the particles [65]. In the following studies, the researchers developed an admixture from the polymerization of a sodium lignosulfonate with poly(ethylene oxide) [72]. The product was compared to chemical admixtures produced from the polymerization of Kraft lignins and sodium lignosulfonate with poly(methacrylic acid) and 3-sulfopropyl methacrylate [73]. As a result, the poly(methacrylic acid)-modified lignins were the most efficient superplasticizers in the Slump test, with similar performance to a commercial polycarboxylate-based superplasticizer.

The studies conducted by Qiu, Zheng, and collaborators should also be noted [18,59,76,77]. The researchers showed that the incorporation of quaternized [76] and polyethylene glycol-modified [77] lignins enhanced the plasticizing performance of a polycarboxylate-based superplasticizer in clay-contained cement pastes. This occurs because the modified lignins are preferentially adsorbed on the montmorillonite surface, enhancing the clay tolerance of the commercial superplasticizer. In the following studies, the researchers developed superplasticizers from polymerization strategies involving Kraft lignins and poly (ethylene oxide) [18,59]. The chemical admixture produced by Zheng et al. [59] was classified as lignin-based polycarboxylates. These authors reported that the side chains of poly(ethylene oxide) promoted steric repulsion between the cement particles, which contributed to the similarity of the plasticizing mechanism between the lignin-based polycarboxylates and commercial polycarboxylate-based superplasticizers.

Yamada, Uraki, and collaborators developed LBAs from different technical lignins [28,74,75]. Aso et al. [74] observed that Kraft lignins modified with different poly(ethylene glycol) derivatives promoted better workability in cement pastes than a commercial lignosulfonate. In this study, softwood lignins showed better efficiency than hardwood lignins. The admixtures containing smaller poly(ethylene glycol) chains showed better dispersion properties, although the authors did not fully explain the phenomenon.

In the following studies, Takahashi et al. [28,75] investigated unmodified Soda lignins and Soda lignins modified by reactions with poly(ethylene glycol) derivatives. The unmodified lignins (with low molecular weight and high phenolic hydroxyl group content) showed plasticizing ability better than a commercial lignosulfonate [28]. The poly(ethylene glycol)-modified lignins presented a performance superior to the unmodified lignins [75]. The authors related this performance to the polyethylene glycol content and the length of the poly(ethylene glycol) chains, which possibly contributed to a higher steric hindrance and a higher phenolic hydroxyl group content in the admixtures produced [75].

Other lignin modification strategies were identified in the literature. Magina et al. [17] adopted different chemical modification strategies to improve the performance of purified lignosulfonate as a plasticizer for concrete mixtures. These authors verified that the most promising products were poly(propylene oxide)-modified lignosulfonate, which improved the dispersing properties of the purified lignosulfonate by approximately 19%. Ji et al. [80] produced LBAs from the copolymerization and blending of sulfanilic acid-phenol-formaldehyde condensates with a commercial sodium lignosulfonate. As with the poly(propylene oxide)-modified lignosulfonate, the mechanism of action of the admixtures produced by copolymerization and blending possibly involved steric hindrance and electrostatic repulsion [17,80]. In another study, Zhang et al. [25] developed LBAs through a blending of a commercial polycarboxylate-based superplasticizer and a sulfate pulping black liquor, followed by modification reactions involving ferrous sulfate, hydrogen peroxide, and sodium sulfite. These LBAs incorporations reduced the mixing water by up to 30.3% compared to a reference mortar, while the commercial polycarboxylate-based superplasticizer promoted a mixing water reduction by up to 30.8%, indicating a similar performance.

### 4.2. Other Properties of New LBAs

#### 4.2.1. Influence on Setting Time and Air Entrainment

Some of the recently developed LBAs were classified as set retarders [19,78,79]. Moreira et al. [19] evaluated a carboxymethylated Soda lignin as a cement set retarder admixture for oilwell application. As a result, the dosages of 4.7, 8.7, and 13.4 L/m³ (liter per cubic meter) of the carboxymethyl lignin provided increases of 17, 66, and 104% in the setting time of the cement pastes, respectively. Li et al. [79] evaluated the retarding performance of diethanolamine-modified lignin. When added at a dosage of 0.05 wt.%, this admixture increased by approximately 50 and 70% the initial and final setting times of cement paste, respectively. The authors attributed this behavior to steric hindrance of molecular structure and electrostatic interactions of anchoring groups adsorbed on the cement particles during the hydration process, such as hydroxyl and amino groups [79].

In another study, Cong et al. [78] developed a set retarder modifying a sodium lignosulfonate by a copolymerization process involving 2-acrylamido-2-methyl propane sulfonic acid and itaconic acid. The adsorption of the modified-lignosulfonate on the solid phases of the mixture contributed to the retarding effect enhancing the workability of modified phosphoaluminate cement pastes with a water/cement ratio of 0.5. In the study conducted by El-Mekkawi et al. [27], the black liquor investigated as a plasticizer was also classified as a set retarder. The incorporation of 30% of LBA in replacement of the water volume increased the initial and final setting times of the cement paste by 223% and 258%, respectively. The authors attributed the retarding performance of the black liquor to the high content of sugars in its composition [27].

The air-entraining capacity of LBAs was also investigated. Zheng et al. [20] reported that amination-modified alkaline pulping black liquor act as an air-entraining admixture in ready-mixed wet mortars. According to the authors, the amino groups introduced into the lignin structure can form strong hydrogen bonds with water, which increases foam wall strength and provides stability and uniformity to the matrix [20]. Air entrainment properties were also observed in LBAs classified as plasticizers or superplasticizers. Kalliola et al. [70] found that incorporating 0.4 wt.% of oxidized lignin led to matrices with an air content of up to 15%, while unmodified lignin matrices presented an air content of approximately 7%. Additionally, air-entraining properties were verified in sulfomethylated lignins [31], poly(ethylene oxide)-modified lignins [59], and unmodified lignins [86].

#### 4.2.2. Influence on Mechanical Properties

A high air content can considerably reduce the compressive strength of cement-based composites [20,70]. The negative influence on the mechanical performance is one of the main disadvantages of using commercial lignosulfonates as cement admixtures [11,91]. However, when properly dosed, some LBAs can reduce the mixing water and promote moderate air entrainment into the cement-based composite, enhancing its mechanical properties [20,27,31,58,82]. Furthermore, using anti-foam admixtures is an alternative to reduce air entrainment in LBA-containing mortars and concretes [70,78].

In addition to reducing cement consumption, the incorporation of mineral admixtures can enhance the mechanical properties of LBA-containing cement-based composites. Unmodified lignins showed good compatibility with meta-aluminate to produce a clay-cement composite grouting material [83]. The good performance of LBAs in cement-based composites containing montmorillonite clay [76,77] and kaolinitic clay with zeolite clinoptilolite [66] was also reported in the literature. In the study conducted by El-Mekkawi et al. [27], the addition of up to 5 wt.% of silica fume enhanced the mechanical performance of concretes containing residual black liquor without affecting their workability. Finally, in hybrid admixtures, the unmodified Kraft lignin was responsible for improving the dispersion of the mineral admixtures (zinc oxide, silica and alumina), which tend to particle agglomeration [84,86,87]. Due to the filler and pozzolanic effects of the mineral admixtures, the mechanical strength of the matrices increased or preserved, depending on the inorganic/organic ratios adopted [84,86]. The researchers also found that the zinc oxide-enriched hybrid admixtures showed high antimicrobial performance [87].

### 4.3. Knowledge Gaps and Considerations for Further Research

Using residual lignins to develop more eco-efficient products is a relevant strategy for emerging economies, especially those with large biomass availability, such as Brazil and India, which have great potential for producing LBAs. The LBAs manufacture contributes to recovering residual lignins from different industrial processes. In addition, the use of LBAs aids in mitigating greenhouse gas emissions, especially because these compounds replace commercial admixtures produced with raw materials from the petrochemical sector.

**Figure 8:**
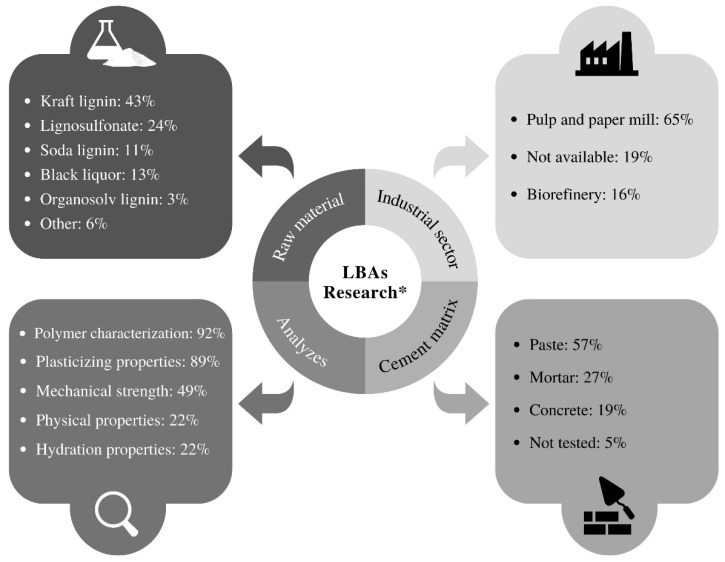
Comparative profile of the content of articles on LBAs (* The data indicate the frequency with which the parameters were adopted in the selected articles).

Figure 8 shows a comparative profile of the content of the 37 articles selected for the qualitative discussion. The authors separate the information related to the type and source of the technical lignins used to produce the LBAs (raw material and industrial sector) and the characteristics of the experimental procedures performed (cement matrix and analyzes).

Results show that at least 65% of the studies on new LBAs used lignins generated in pulp and paper mills. Biorefineries lignins, in turn, need higher valorization in this research area. Previous studies demonstrated the feasibility of producing chemical admixtures from lignocellulosic biomass generated in biorefineries [28,31,69]. In this sense, the LBAs research can offer fundamental strategies for waste management in biorefineries, considering the high availability of biomass jointly with the increasing development of biofuels in the industry [45].

As expected, Kraft lignin is the most studied technical lignin in LBAs research. On the other hand, Organosolv lignins still need further studies on their modification and improvement of surfactant properties. Due to their high purity and the feasibility of being generated from a sustainable delignification process, Organosolv lignins are becoming popular and valued in the global market [3,8]. Thus, developing new LBAs from Organosolv lignins generated by green chemistry approaches in the context of biorefineries is a research topic that needs to be explored.

The use of residual black liquors is another strategy for producing new LBAs. This strategy aids in avoiding the improper disposal of black liquors and reduces costs in LBAs production since fewer modification processes are necessary. Previous studies used residual black liquors from different delignification processes to produce LBAs [20,25,68]. However, Figure 8 shows that these studies correspond to only 13% of the published articles on new LBAs, demonstrating that there is still room for further research.

The LBAs research has a multidisciplinary character and must involve different tests and analyses. The science mapping allowed the identification of various journals related to civil engineering that publish on LBAs (see Table 1). However, most of the works involve commercial lignosulfonate-based admixtures. Discussions about the production and testing of new LBAs are more frequent in chemical engineering, materials engineering, and agricultural sciences journals. As a possible consequence, 92% of the studies on new LBAs performed a polymer characterization, while only 49% investigated the mechanical strength and 22% investigated the physical properties of LBA-containing composites. Mechanical and physical analyses of hardened cement matrices are essential to verify the feasibility of using LBAs and should be considered for near-future research on LBAs applied to cement-based composites. In addition, Figure 8 shows that only 22% of the studies on new LBAs analyzed the hydration properties of the cement matrices. Investigations on the hydration profile of LBA-containing composites (e.g., calorimetric and setting time analyses) are mandatory to assess the influence of LBAs on cement hydration since LBAs may present set-retarding properties.

Results show that only 46% of the studies on LBAs were performed in concrete and mortar. Investigating the performance of cement-based composites containing aggregates brings the research closer to the practical applications of cement matrices. In addition, LBAs applications can expand toward developing LBA-containing special concrete and mortar. Some LBAs with air-entraining properties can be used to produce lightweight matrices (together with different lightweight aggregates).

In addition, nanotechnology in cement-based composites has been a growing research topic in recent years. Incorporating nanomaterials (e.g., nano silica and nano alumina) in the cement matrix is a strategy for producing ultra-high performance concrete (UHPC) since they enhance mechanical performance [92,93,94]. Previous studies reported that incorporating mineral admixtures enhances the fresh and hardened properties of LBA-containing composites. [27,84,86]. Thus, an alternative to increasing the eco-efficiency of UHPCs for further research is to incorporate hybrid admixtures, suspensions, or mixtures composed of LBAs and residual mineral admixtures at a nanoscale, such as ground-granulated blast furnace slag and rice husk ash.

The science mapping and qualitative discussion presented valuable information about the research on new LBAs. Comprehending the current research scenario highlights new opportunities for early-stage researchers, industry professionals, and funding authorities. Thus, developing new LBAs is a crucial step toward consolidating more sustainable practices in the construction industry, mainly in countries with high biomass availability.

## 5. Conclusions

The present study holistically approached the global LBAs research applied to cement-based composites. Lignin valorization has gained popularity since 2011 and it is expected that more near-future studies will be published in this area. The scientometric analysis revealed that China was the most collaborative country in the LBAs research, although the USA and Canada also collaborated significantly. In addition, the LBAs research encompasses journals with different categories (e.g., agricultural and biological sciences and materials science), demonstrating the multidisciplinarity of the subject.

The in-depth analysis of the literature data indicated that there is still room for further research on LBAs. In summary, the qualitative discussion revealed that:

- Most research has focused on developing LBAs using modified and unmodified Kraft lignins. The use of other technical lignins must be better understood to expand and diversify the LBAs research;
- The use of lignins obtained in biorefinery processes and residual black liquors is a viable alternative to consolidate emerging economies in LBAs research. In addition, incorporating LBAs jointly with residual mineral admixtures is an important step toward more sustainable practices in the construction industry;
- LBAs can be classified as plasticizers, superplasticizers, air-entraining agents, and set retarders. The LBAs developed so far were applied mainly in ordinary cement-based composites. In this sense, there is a lack of studies focusing on the incorporation of LBAs in special concrete and mortar (e.g., UHPC and lightweight concrete);
- Most research focused on the production processes and chemical characterization of LBAs. However, the evaluation of the mechanical and physical properties of LBA-containing composites needs more attention in further research since unwanted side effects of LBAs may affect the matrix-hardened properties.

It stands out that the authors dealt with the limitations of the database and the software used. To conclude, it is expected that the innovation of this study will contribute to the understanding of lignin-based admixtures and the discussion on the development of more sustainable cement-based composites.

## Figures and Tables

**Table 1:** Statistical analysis of journals in the LBAs research.

Journal Source	No. of Articles	Citations	Total Link Strength	Average Citations	Av. Norm. Citations
*Construction and Building Materials*	14	292	6	20.86	1.53
*Cement and Concrete Research*	10	186	16	18.60	1.54
*ACS Sustainable Chemistry and Eng.*	9	207	15	23.00	1.41
*Advances in Cement Research*	8	25	5	3.13	0.18
*Cement and Concrete Composites*	5	330	2	66.00	1.95
*Industrial Crops and Products*	4	189	6	47.25	1.79
*RSC Advances*	4	58	10	14.50	1.3
*Industrial and Eng. Chemistry Research*	4	92	12	23.00	1.19
*Polymers*	4	45	10	11.25	0.86
*Holzforschung*	4	48	5	12.00	0.56
*Bioresources*	3	116	3	38.67	1.87
*ACI Materials Journal*	3	45	0	15.00	0.77
*Journal of Wood Chemistry and Tec.*	3	47	2	15.67	0.67

**Table 2:** Statistical analysis of keywords studied in the LBAs research.

Keyword	Occurrences	Total Link Strength
Rheology	11	10
Cement *	10	9
Lignin *	13	9
Concrete *	8	8
Adsorption	8	7
Lignosulfonate *	12	7
Admixture *	7	6
Dispersion	7	6
Superplasticizer *	6	6
Workability	7	6
Admixtures *	6	5
Cement paste *	6	5
Plasticizer *	4	4
Superplasticizers *	4	4
Biopolymers	3	3

* Keywords adopted in the bibliometric search.

**Table 3:** Statistical analysis of collaborative scholars in the LBAs research.

Author	Publications	Citations	Average Citations	Av. Norm. Citations	Total Link Strength
Qiu X.	20	336	16.80	0.97	19
Fatehi P.	11	268	24.36	1.41	5
Yang D.	9	208	23.11	1.29	9
Lou H.	6	142	23.67	1.09	6
Pang Y.	6	149	24.83	1.29	6
Zheng D.	6	47	7.83	0.71	5
Justnes H.	5	118	23.60	1.27	3
Konduri M.K.R.	5	154	30.80	1.62	5
Washburn N.R.	5	49	9.80	0.6	4
Zheng T.	5	45	9.00	0.83	5
Bassioni G.	4	17	4.25	0.21	3
Gupta C.	4	46	11.50	0.64	4
Lauten R.A.	4	69	17.25	1.04	3
Uraki Y.	4	57	14.25	0.55	4
Yamada T.	4	57	14.25	0.55	4
Zhou M.	4	64	16.00	1.21	4
Jesionowski T.	4	25	6.25	0.62	3
Klapiszewska I.	4	25	6.25	0.62	3
Klapiszewski L.	4	25	6.25	0.62	3
Ali M.M.	3	17	5.67	0.28	3
Liu W.	3	17	5.67	0.5	1
Pundienė I.	3	24	8.00	0.6	0
Velázquez-Navarro J.F.	3	10	3.33	1.58	0
Wang H.	3	59	19.67	1.39	1

**Table 4:** Statistical analysis of productive countries in LBAs research.

Country	No. of Articles	Citations	Average Citations	Av. Norm. Citations	Total Link Strength
China	44	765	17.39	0.98	6
USA	18	179	9.94	0.67	6
Canada	14	359	25.64	1.50	6
Spain	8	193	24.13	1.65	0
Egypt	7	55	7.86	0.76	4
Norway	7	129	18.43	1.02	0
Brazil	6	105	17.50	0.89	1
Germany	6	70	11.67	0.47	3
Turkey	6	52	8.67	0.72	2
Lithuania	5	29	5.80	0.41	0
Australia	4	285	71.25	1.90	1
India	4	29	7.25	0.31	0
Japan	4	57	14.25	0.55	0
France	3	123	41.00	1.59	2
United Arab Emirates	3	17	5.67	0.28	3

**Table 5:** Statistical analysis of relevant terms in the LBAs research.

Terms	Occurrences	Relevance Score
FTIR	10	4.7954
Molecular weight	21	4.4322
Kraft Lignin *	17	4.0542
Dispersant *	28	3.5232
Suspension *	28	3.3739
Modification	16	2.7296
Preparation	14	2.5526
Zeta Potential	20	2.3811
Hydration	22	1.5207
Lignin *	58	1.4878
Application	28	1.4509
Mechanical property	14	1.4308
Yield Stress	10	1.1772
Compressive strength	20	1.0535
Polycarboxylate	25	0.9914
Plasticizer *	18	0.9477
Workability	18	0.8347
Cement *	54	0.8315
Strength	26	0.7787
Rheology	19	0.7197

* Terms adopted in the bibliometric search.

**Table 6:** Studies on new LBAs for cement-based composites.

Lignin Type and Source	Molecular Weight (g/mol)	Classification	Modification Method	Mineral Admixtures	Dosages	Ref.
Kraft lignin isolated from wheat straw pulping black liquor	25,700	Superplasticizer	Sulfonation	None	0.2, 0.3, 0.4, 0.5, 0.6, 0.8, and 1.0 wt.% of cement	[58]
Alkaline lignin from the pretreatment of bamboo residues	15,470	Plasticizer	Sulfomethylation	Not available	0.1, 0.2, 0.4, 0.6, and 0.8 wt.% of cement	[31]
Hardwood Kraft lignin	53,360	Plasticizer	Sulfomethylation	Not available	1.0 wt.% of cement	[62]
Black liquor from Soda pulping	13,200	Plasticizer	Sulfonation	Fly ash	0.5, 1.0, 1.5, 2.0, 3.0 wt.% of cement	[68]
Not available	Not available	Plasticizer	Sulfomethylation	Not tested	Not tested	[69]
Commercial sodium lignosulfonate	23,650	Superplasticizer	Oxidation and Sulfomethylation	None	0.3 wt.% of cement	[22]
Pinewood Organosolv lignin	15,000 to 22,000	Plasticizer	Oxidation and Sulfomethylation	None	0.3 wt.% of cement	[14]
Softwood Kraft lignin	18,299	Superplasticizer	Oxidation and Sulfomethylation	None	0.2, 0.3, 0.4, 0.5 wt.% of cement	[12]
Wheat straw Soda lignin	3200 to 7320	Plasticizer	Oxidation by O2 in alkaline conditions	None	0.4 wt.% of cement	[70]
Magnesium lignosulfonate	36,800	Plasticizer	Laccase-catalyzed oxidative treatment	Not tested	Not tested	[71]
Kraft lignin	Not available	Superplasticizer	Controlled radical polymerization using polyacrylamide	Kaolin clay and clinoptilolite zeolite	0.5 and 5 mg/mL or 0.025, and 0.25 wt.%, respectively	[66]
Kraft lignin	Not available	Superplasticizer	Controlled radical polymerization using polyacrylamide	None	0.025, 0.05, 0.1, 0.15, 0.2, and 0.25 wt.% of cement	[65]
Kraft lignin and sodium lignosulfonate	10,000	Superplasticizer	Controlled radical polymerization using polyacrylamide	Simulation of cement paste with magnesium oxide suspensions	0.25 and 2.7 mg/mL	[67]
Commercial sodium lignosulfonate	20,000	Superplasticizer	Polymerization using poly(ethylene oxide)	None	0.3, 0.4, and 0.5 wt.% of cement	[72]
Kraft lignin and commercial sodium lignosulfonate	2300 to 3880	Superplasticizer	Radical polymerization using poly(methacrylic acid) and poly(3-sulfopropyl methacrylate)	None	0.25 wt.% of cement	[73]
Pinewood Kraft lignin	149,100 to 192,200	Superplasticizer	Polymerization using poly(ethylene oxide)	None	0.07, 0.09, 0.11, 0.13, and 0.15 wt.% of cement	[59]
Pinewood Kraft lignin	7400 to 22,300	Superplasticizer	Polymerization using poly(ethylene oxide)	None	0.1 and 0.5 wt.% of cement	[18]
Kraft lignin and black liquor from hardwood and softwood Kraft pulping	Above 3000	Plasticizer	Modifications using poly(ethylene glycol) derivatives	None	0.2, 0.4, 0.6, and 0.8 wt.% of cement	[74]
Soda lignin isolated from Japanese cedar pulping black liquor	3600 to 9100	Plasticizer	No modification	None	0.2, 0.4, and 0.6 wt.% of cement	[28]
Soda lignin isolated from Japanese cedar pulping black liquor	Not available	Plasticizer	Modifications using Poly(ethylene glycol) derivatives	None	0.2, 0.4, 0.6, 0.8, and 1.2 wt.% of cement	[75]
Purified lignosulfonate from acid magnesium-based sulfite pulping of eucalyptus	3240	Plasticizer	Different modification methods, including modifications with poly(ethylene glycol) and poly(propylene glycol) derivatives	None	2.5 wt.% of cement of an admixture solution containing 40 wt.% solids content	[17]
Pinewood lignosulfonate	12,000	Anti-Slurry	Quaternization using 3-chloro-2-hydroxypropyl trimethylammonium chloride	Montmorillonite clay	0.01 wt.% of cement	[76]
Pinewood Kraft lignin	18,700	Anti-Slurry	Polymerization using polyethylene glycol	Montmorillonite clay	0.01, 0.02, 0.03, and 0.04 wt.% of cement	[77]
Commercial sodium lignosulfonate	Not available	Set retarder	Copolymerization using 2-acrylamido-2-methyl propane sulfonic acid and itaconic acid	None	0.4, 0.5, 0.6, 0.7, and 0.8 wt.% of cement	[78]
Soda lignin isolated from sugarcane bagasse pulping black liquor	Not available	Set retarder	Carboxymethylation	None	4.68, 8.69, and 13.37 L/m³ (liter per cubic meter of cement)	[19]
Not available	Not available	Grinding aid and set retarder	Reaction with diethanolamine	None	0.01, 0.02, 0.03, 0.04, and 0.05 wt.% of cement	[79]
Black liquor from rice straw Soda pulping	Not available	Plasticizer and set retarder	No modification	Silica fume	5, 15, 25, and 35% in replacement of the water volume	[27]
Black liquor from sugarcane bagasse alkaline pulping	Not available	Air-entraining	Amination through Mannich reaction	Fly ash	0.1, 0.2, 0.3, 0.4, and 0.5 wt.% of cement	[20]
Commercial sodium lignosulfonate	24,180 to 31,890	Superplasticizer	Copolymerization and blending using sulphanilic acid–phenol–formaldehyde condensates	None	0.6, 0.8, 1.0, and 1.2 wt.% of cement	[80]
Black liquor from wheat stalk sulfate pulping	Not available	Superplasticizer	Mixing with polycarboxylate-based superplasticizer and modification processes using polycarboxylate-based superplasticizer, ferrous sulfate, hydrogen peroxide and sodium sulfite	None	0.35, 0.4, 0.45, and 0.5 wt.% of cement	[25]
Lignosulfonate isolated from corncob residue sodium sulfite pretreatment	Not available	Plasticizer	No modification	Not tested	Not tested	[81]
Deep eutectic solvent lignins isolated from sugarcane bagasse and coffee chaff	Not available	Plasticizer	No modification/Carboxymethylation	None	0.3, 2.0, and 4.0 wt.% of cement	[82]
Not available	Not available	Plasticizer	No modification	Clay and meta-aluminate	1.0, 1.5, 2.0, and 3.0 wt.% of cement	[83]
Industrial Kraft lignin and commercial magnesium lignosulfonate	10,000	Hybrid admixture	High energy milling between the inorganic-organic materials	Alumina	0.25, 0.5, and 1.0 wt.% of cement, with alumina/lignin ratios of 5, 2, 1, 0.5, and 0.2 (wt./wt.)	[84,85]
Industrial Kraft lignin	10,000	Hybrid admixture	High energy milling between the inorganic-organic materials	Silica	0.5 and 1.0 wt.% of cement, with silica/lignin ratios of 5, 2, 1, 0.5, and 0.2 (wt./wt.)	[86]
Industrial Kraft lignin	Not available	Hybrid admixture	High energy milling between the inorganic-organic materials	Zinc oxide and Silica	0.1 wt.% of cement, with zinc oxide/lignin and zinc oxide-silica/lignin ratios of 5,1 and 0.2 (wt./wt.)	[87]

## Data Availability

The data used in this research have been properly cited and reported in the main text.
